# The India–Asia collision system

**DOI:** 10.1093/nsr/nwaf487

**Published:** 2025-11-07

**Authors:** Jie Yuan, Chenglong Deng, Zhenyu Yang, Wout Krijgsman

**Affiliations:** State Key Laboratory of Lithospheric and Environmental Coevolution, Institute of Geology and Geophysics, Chinese Academy of Sciences, China; State Key Laboratory of Lithospheric and Environmental Coevolution, Institute of Geology and Geophysics, Chinese Academy of Sciences, China; College of Earth and Planetary Sciences, University of Chinese Academy of Sciences, China; College of Resources, Environment and Tourism, Capital Normal University, China; Department of Earth Sciences, Utrecht University, The Netherlands

The collision between the Indian and Asian continents and the continued indentation of India into Asia have resulted in the formation of the Himalayan orogen and played a pivotal role in the uplift of the Tibetan Plateau [1, 2]. Reconstructing the paleogeography of this collision system is fundamental for advancing our understanding of plate tectonic processes, oceanic circulation patterns, climate change and biodiversity evolution through geological time [3–5].

As the most prominent example of continent–continent collision on Earth, the Himalayan orogen offers a unique opportunity to study orogenic processes. Since the onset of collision, ongoing convergence has caused intense crustal shortening, large-scale faulting and significant erosion across the orogen. Despite extensive research, several aspects of the India–Asia collision system remain debated, including the size of Greater India, the possible existence of a newly formed ocean basin and the presence of a Trans-Tethyan subduction zone within the Neo-Tethys Ocean. To address these issues, at least five competing tectonic models have been proposed: the single-stage large Greater India collision model (Fig. 1a) [6], three two-stage India–Asia collision models [the island arc-continent model (Fig. 1b) [7,8], the Greater India Basin model (Fig. 1c) [9] and the Xigaze back-arc basin model (Fig. 1d) [10]] and a triple-stage model involving arc–continent collision and subsequent two-stage continent–continent collision (Fig. 1g–j) [11,12].

**Figure 1.**
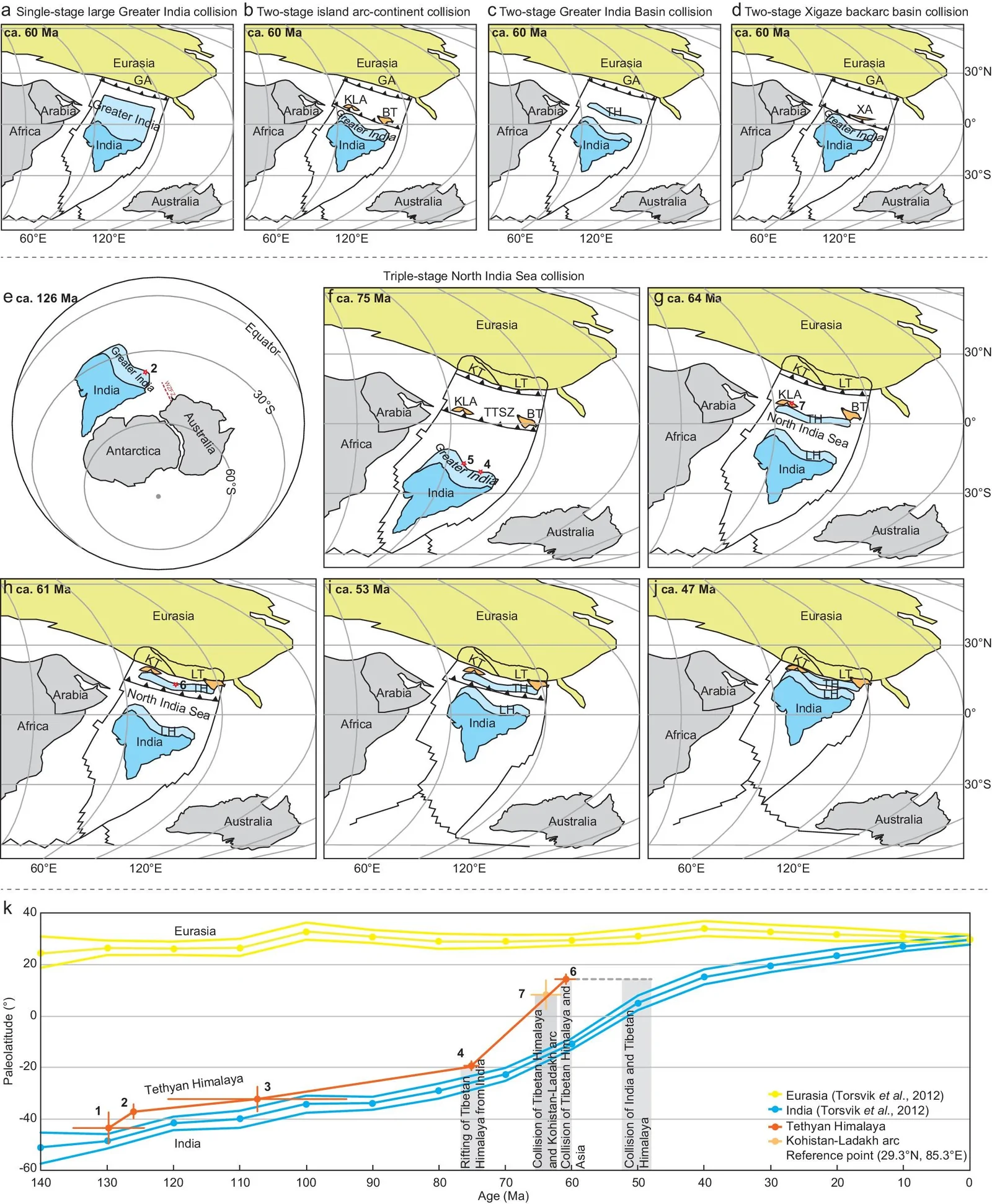
(a−d) Hypotheses for the single-stage large Greater India collision model [6] and the two-stage India–Asia collision models, including the island arc–continent model [7,8], Greater India Basin model [9] and Xigaze back-arc basin model [10]. (e−j) Paleogeographic evolution of the India–Asia collision system from ca. 126 Ma to ca. 47 Ma associated with the triple-stage North India Sea model [11,12]. (k) Early Cretaceous−Paleocene paleomagnetic data from the northern subzone of the Tethyan Himalaya and from the Kohistan–Ladakh arc on the Eurasian and Indian paleolatitude versus time curves, using the apparent polar wander paths [13]. The red stars in (e−h) indicate the observed paleolatitudes from the northern subzone of the Tethyan Himalaya and from the Kohistan–Ladakh arc (see the Supplementary data for details of each pole). GA, Gangdese arc; LT, Lhasa terrane; KT, Karakoram terrane; KLA, Kohistan–Ladakh arc; XA, Xigaze arc; BT, Burma terrane; TH, Tibetan Himalaya; LH, Lesser Himalaya; TTSZ, Trans-Tethyan subduction zone; WZFZ, Wallaby–Zenith fracture zone.

The size of Greater India is a key factor in the India–Asia collision system and exerts a significant influence on the timing and geometry of the collision process. In recent years, multiple groups have conducted paleomagnetic investigations on lava flows, limestones and oceanic red beds from the northern subzone of the Tethyan Himalaya, which represents the most distal portion of the Indian passive continental margin. These high-quality paleomagnetic datasets provide robust paleolatitudinal constraints, placing the Tethyan Himalaya at approximately 43.5°S at ∼130 Ma, 37.2°S at ∼126 Ma, 32.3°S at ∼107 Ma, and 19.4°S at ∼75 Ma for the reference point (29.3°N, 85.3°E) (Table S1) (Fig. 1k). Comparison of these paleolatitudes, with the expected positions of India based on the apparent polar wander path of Torsvik *et al.* [13], indicates that Greater India extended nearly N–S by ∼900 km between ∼130 and 75 Ma (Fig. 1k). This relatively small-sized Greater India is consistent with geological, seismological and geochemical interpretations [7,10,12].

The existence of a newly formed ocean basin constitutes a critical issue within the India–Asia collision system. van Hinsbergen *et al.* [9] proposed that rifting of the Tibetan Himalaya (Tethyan Himalaya plus Greater Himalaya) from the Indian continental margin led to the formation of an intervening ocean basin between ∼120 and 65 Ma. In contrast, Kapp and DeCelles [10] argued that rifting of the Xigaze forearc from the Asian margin generated a back-arc ocean basin between ∼90 and 70 Ma. Our recent paleomagnetic data constrain the Tethyan Himalaya to paleolatitudes of 19.4°S at ∼75 Ma and 14.1°N at ∼61 Ma (Fig. 1k) [11,12] and indicate that the Tibetan Himalaya drifted northward at an average speed of ∼26 cm/year, significantly faster than India’s drift speed of ∼11 cm/year over the same time interval (75−61 Ma). These observations led us to hypothesize that the Tibetan Himalaya rifted away from India between ∼75 and 61 Ma, generating an ocean basin referred to as the North India Sea (Fig. 1f–k) [11,12]. This rifting may have been triggered by the melting of the lower portion of the Indian passive continental margin lithosphere, associated with the upwelling of the Réunion plume [14].

Based on paleomagnetic data, Martin *et al.* [8] constrained the Kohistan–Ladakh arc to a paleolatitude of 8.1°N at ∼64 Ma, placing the Trans-Tethyan subduction zone >1000 km south of the Asian margin. Integrating this arc-continent collision with the North India Sea scenario [11] resulted in the triple-stage India–Asia collision model involving an arc–continent collision followed by two-stage continent–continent collision (Fig. 1g–j) [12]. The first collision between the Tibetan Himalaya and the intra-oceanic island arc (e.g. the Kohistan–Ladakh arc and the Burma terrane) occurred at ∼64 Ma. The second collision, involving the Tibetan Himalaya (including the accreted island arc) and the Lhasa terrane, took place along the Shyok–Yarlung Tsangpo suture zone at ∼61 Ma. The final collision between India and the Tibetan Himalaya occurred along the Main Central Thrust during 53–47 Ma.

The triple-stage collision scenario reconciles multidisciplinary evidence from geology, geochemistry, geophysics and paleontology. It is also consistent with the India–Asia convergence rates inferred from the relative plate motions between India–Africa and India–Antarctica [14]. Nevertheless, the controversy regarding the India–Asia collision process is expected to persist. For example, numerical modelling suggests that the lithosphere subducted north of the Indian subcontinent may have been a relatively young and buoyant oceanic lithosphere [15], potentially associated with the North-India-Sea oceanic lithosphere [11,12]. However, the absence of ophiolites between the Tethyan Himalaya and Lesser Himalaya casts doubts on the existence of a newly formed ocean basin. Therefore, detailed geological investigations along the Main Central Thrust are critical for resolving these outstanding questions. In addition, acquiring more high-quality paleomagnetic data from the Tethyan Himalaya is essential for reconstructing its kinematic history with greater precision. Together, these approaches will further enhance our understanding of the tectonic dynamics governing the India–Asia collision system.

In summary, we emphasize that the India–Asia collision system is highly complex, encompassing at least a relatively small-sized Greater India, a newly formed ocean basin and a Trans-Tethyan subduction zone in the Neo-Tethys Ocean. Combined with additional geological, geochemical and geophysical investigations of the Himalayan orogen [12], it has become clear that multiple collision events occurred in the India–Asia orogenic system. Nonetheless, all theoretical frameworks must be rigorously validated through empirical testing and practical application. Likewise, the India–Asia collision model outlined in this Perspective will be subject to ongoing evaluation and refinement through the integration of multidisciplinary evidence and cross-disciplinary validation.

## Supplementary Material

nwaf487_Supplemental_File
