# Supplementary material for: The India–Asia collision system
Source: Natl Sci Rev. 2025 Nov 7;13(17):nwaf487. doi: 10.1093/nsr/nwaf487 (PMC13552060; doi:10.1093/nsr/nwaf487)
Supplement: Supplementary file 1 [file nwaf487_supplemental_file.pdf]

Supplementary Materials for

The India-Asia collision system

Jie Yuan[1], Chenglong Deng[1,2,*], Zhenyu Yang[3] and Wout Krijgsman[4]

[1]State Key Laboratory of Lithospheric and Environmental Coevolution, Institute of Geology and Geophysics, Chinese Academy of Sciences, Beijing 100029, China;
[2]College of Earth and Planetary Sciences, University of Chinese Academy of Sciences, Beijing 100049, China;
[3]College of Resources, Environment and Tourism, Capital Normal University, Beijing 100048, China;
[4]Department of Earth Sciences, Utrecht University, Utrecht HD 3584, The Netherlands

*Corresponding author. Email: cldeng@mail.iggcas.ac.cn

This file includes the details of each pole from the northern subzone of the Tethyan Himalaya and from the Kohistan-Ladakh arc.

Supplementary Table 1. Early Cretaceous-Paleocene paleomagnetic data from the northern subzone of the Tethyan Himalaya and from the Kohistan-Ladakh arc

ID	Site (°N/°E)	Time (Ma)	N/n	Paleopole			Paleolat. (°)	Rock type	Reliability criteria Q	Inclination shallowing estimation	Rock magnetic analysis	Reference
				Plat. (°)	Plon. (°)	A_{95} or (dp/dm)						
**Tethyan Himalaya**					**At Reference Position (29.3°N, 85.3°E)**							
1	28.8/91.3	135.1–124.4	26/216	-5.9	308.0	6.1	-43.5 ± 6.1	lava	7	-	+	Ma et al. (2016)
2	28.9/91.1	~126.0	-/204	11.2	300.5	2.6	-37.2 ± 2.6	limestone	6	E/I	+	Yuan et al. (2025)
3	29.7/84.0	120.8–93.9	-/53	25.0	285.7	4.8/7.1	-32.3 ± 4.8	red bed	7	A	+	Qin et al. (2019)
4	28.9/89.2	76.2–74.0	-/127	40.8	256.3	1.8	-19.4 ± 1.8	red bed	7	E/I, A	+	Yuan et al. (2021)
5	29.8/83.6	~75.0	-/116	43.3	258.3	1.8	-17.1 ± 1.8	red bed	6	E/I, A	+	Yuan et al. (2023)
6	29.3/85.3	62.5–59.2	-/162	74.8	263.4	1.9	14.1 ± 1.9	red bed	7	A	+	Yuan et al. (2022)
**Kohistan-Ladakh arc**												
7	34.4/77.7	66–62	18/-	64	266.4	5.6	8.1 ± 5.6	lava	7	-	+	Martin et al. (2020)

Abbreviations are: ID, Serial number; n/N, number of samples or sites used to calculate Fisher mean; Plat. and Plong. are latitude and longitude of paleopoles; A_{95} is the radius of cone at 95% confidence level about the mean paleopole, A_{95} = (dp*dm)^0.5; Paleolat. is the paleolatitude calculated for the reference point at 29.3°N, 85.3°E; Data reliability criteria is after: 1, well-determined rock age and a presumption that magnetization is the same age; 2, sufficient quantity of samples (n > 50 specimens or N > 5 sites) and adequate statistical precision, k (or K) ≥ 10 and α_{95} (A_{95}) ≤ 16.0; 3, detailed demagnetization and isolation of magnetic components; 4, positive field stability tests; 5, structural control and tectonic coherence with craton or block involved; 6, presence of reversals; 7, no resemblance to paleopoles of younger age (by more than a period) (Van der Voo, 1990). Q, the reliability factor; Inclination shallowing correction: E/I, E/I inclination shallowing correction; A, anisotropy-based inclination correction; Rock magnetic analysis,“+” means fulfilling this criterion; “-”means failing to fulfill this criterion.

1. Ma Y, Yang T and Bian W et al. Early Cretaceous paleomagnetic and geochronologic results from the Tethyan Himalaya: Insights into the Neotethyan paleogeography and the India–Asia collision. Sci Rep 2016; 6: 21605.
2. Martin CR, Jagoutz O and Upadhyay R et al. Paleocene latitude of the Kohistan-Ladakh arc indicates multistage India-Eurasia collision. Proc Natl Acad Sci USA 2020; 117: 29487–29494.
3. Qin SX, Li YX and Li XH et al. Paleomagnetic results of Cretaceous cherts from Zhongba, southern Tibet: New constraints on the India-Asia collision. J Asian Earth Sci 2019; 173: 42–53.
4. Van der Voo R. The reliability of paleomagnetic data. Tectonophysics 1990; 184: 1–9.
5. Yuan J, Deng C and Yang Z et al. New paleomagnetic data from the central Tethyan Himalaya refine the size of Greater India during the Campanian. Earth Planet Sci Lett 2023; 622: 118422.
6. Yuan J, Deng C and Yang Z et al. Triple-stage India-Asia collision involving arc-continent collision and subsequent two-stage continent-continent collision. Glob Planet Change 2022; 212: 103821.
7. Yuan J, Hao W and Qiu R. New paleomagnetic and geochronologic results from the eastern Tethyan Himalaya refine the size of Greater India in the Early Cretaceous. J Asian Earth Sci 2025; 278: 106428.
8. Yuan J, Yang Z and Deng C et al. Rapid drift of the Tethyan Himalaya terrane before two-stage India-Asia collision. Natl Sci Rev 2021; 8: nwaa173.
